# Supplementary material for: Vaccination induces rapid protection against bacterial pneumonia via training alveolar macrophage in mice
Source: eLife. 2021 Sep 20;10:e69951. doi: 10.7554/eLife.69951 (PMC8455131; doi:10.7554/eLife.69951)
Supplement: Supplementary file 1. [file elife-69951-supp1.docx]

**Table S1. Primers used in real-time PCR.**

| Primer name | Primer sequence |
| --- | --- |
| CXCL1-Forward | 5’-ATGGCTGGGATTCACCTCAA-3’ |
| CXCL1-Reverse | 5’-AGTGTGGCTATGACTTCGGT-3’ |
| CXCL2- Forward | 5’-AGGGCGGTCAAAAAGTTTGC-3’ |
| CXCL2- Reverse | 5’-CAGGTACGATCCAGGCTTCC-3’ |
| CXCL5- Forward | 5’-TGGCATTTCTGTTGCTGTTC-3’ |
| CXCL5- Reverse | 5’-CACCTCCAAATTAGCGATCAA-3’ |
| CXCL10- Forward | 5’-ATCATCCCTGCGAGCCTATCCT-3’ |
| CXCL10- Reverse | 5’-GACCTTTTTTGGCTAAACGCTTTC-3’ |
| CCL2- Forward | 5’-TTAAAAACCTGGATCGGAACCAA-3’ |
| CCL2- Reverse | 5’-GCATTAGCTTCAGATTTACGGGT-3’ |
| CCL7- Forward | 5’-CCACCATGAGGATCTCTGC-3’ |
| CCL7- Reverse  IL-6- Forward  IL-6- Reverse  TNF-α- Forward  TNF-α- Reverse | 5’-TTGACATAG CAGCATGTGGAT-3’  5′-CCTCTCTGCAAGAGACTTCC-3’  5′-CTCCGGACTTGTGAAGTAGG-3’  5’- CCTATGTCTCAGCCTCTTCTCAT-3’  5’- CACTTGGTGGTTTGCTACGA-3’ |
| β-actin- Forward | 5’-GGCTGTATTCCCCTCCATCG-3’ |
| β-actin- Reverse | 5’-CCAGTTGGTAACAATGCCATGT-3’ |
